# Cumulative revision rates in total hip arthroplasty depending on acetabular cup fixation: an analysis from the German arthroplasty registry

**DOI:** 10.1186/s42836-026-00420-8

**Published:** 2026-08-06

**Authors:** Alberto Alfieri Zellner, Alexander Grimberg, Yinan Wu, Mari Babasiz, Frank Sebastian Fröschen, Dieter Christian Wirtz

**Affiliations:** 1 Department of Orthopedics and Trauma Surgery, University Hospital Bonn, Bonn, 53127 Germany; 2 EPRD Deutsche Endoprothesenregister gGmbH, Berlin, 10623 Germany

**Keywords:** Total hip arthroplasty, National joint registry, Acetabular cup fixation, Cemented fixation, Cementless fixation, Revision-free survival, Kaplan–Meier, Cox proportional hazards

## Abstract

**Background:**

While modern third-generation cementation techniques have optimized outcomes for cemented fixation, cementless acetabular fixation is currently the widely preferred cup option in Germany. In elderly patients, however, international registry data suggest a potential advantage for cemented acetabular fixation strategies. Given the rising volume of total hip arthroplasties (THA) and an ageing population, we performed a registry-based analysis to evaluate revision risk associated with the type of acetabular fixation in elderly patients.

**Methods:**

A registry-based cohort analysis of 330,910 primary THAs was performed. The overall median follow-up was 3.48 years (IQR 1.52–5.82), with a mean follow-up of 3.79 years (SD 2.60) and a maximum follow-up duration of 11.38 years. Statutory health insurance billing data, implant manufacturer product database, and hospital electronic case report forms were evaluated. Longitudinal follow-up was conducted for all-cause revisions and mortality. Estimated risk of revision for any reason at eight years was assessed using Kaplan–Meier, adjusting for age and femoral stem fixation (cemented vs cementless). Furthermore, multivariable Cox proportional hazard regression analyses were performed.

**Results:**

Age-stratified analyses showed higher revision rates in patients < 75 years for cemented polyethylene (PE) and highly-cross-linked polyethylene (XPE) cups (both 7.7%) compared to patients ≥ 75 years (5.1% for PE; 4.5% for XPE). In patients ≥ 75 years, the combination of a cemented XPE cup with a cemented stem achieved, together with hybrid THA, the lowest eight-year revision rate (4.1%). Among patients ≥ 80 years (*n* = 63,278), cemented PE/XPE cups had the highest eight-year revision-free survival (95.5%), compared to hemispherical press-fit cups (94.6%) (*p* = 0.015), independent of the type of femoral fixation. Within the ≥ 80-year subgroup with cemented stems, no significant differences were observed between cup types (*p* = 0.100). However, in multivariable Cox regression analyses, cemented XPE cups were associated with revision risks comparable to those of hemispherical cups in patients aged ≥ 75 years with no significant differences (HR 0.98, 95% CI 0.84–1.14; *p* = 0.80) and ≥ 80 years (HR 0.98, 95% CI 0.81–1.19; *p* = 0.80). Within the cemented (X)PE cups subgroup, larger head size, ceramic-on-polyethylene, lower comorbidity burden, and a BMI of ≤ 30 were all significantly associated with improved revision-free survival.

**Conclusion:**

The German arthroplasty registry indicates that, although cementless hemispherical press-fit cups remain the predominant acetabular fixation method used for THA across all age groups, cemented XPE cups have no clinically relevant survivorship differences in selected elderly subgroups (≥ 75-year-old; ≥ 80-year-old patients). For < 75-year-old patients, hemispherical press-fit cups remain the most favorable option. Future studies should focus on prospective analyses of patient-reported outcomes, complication profiles, and long-term cost-effectiveness for acetabular fixation in THA to further define the optimal fixation strategy for different age and risk groups.

## Background

**Fig. 1:**
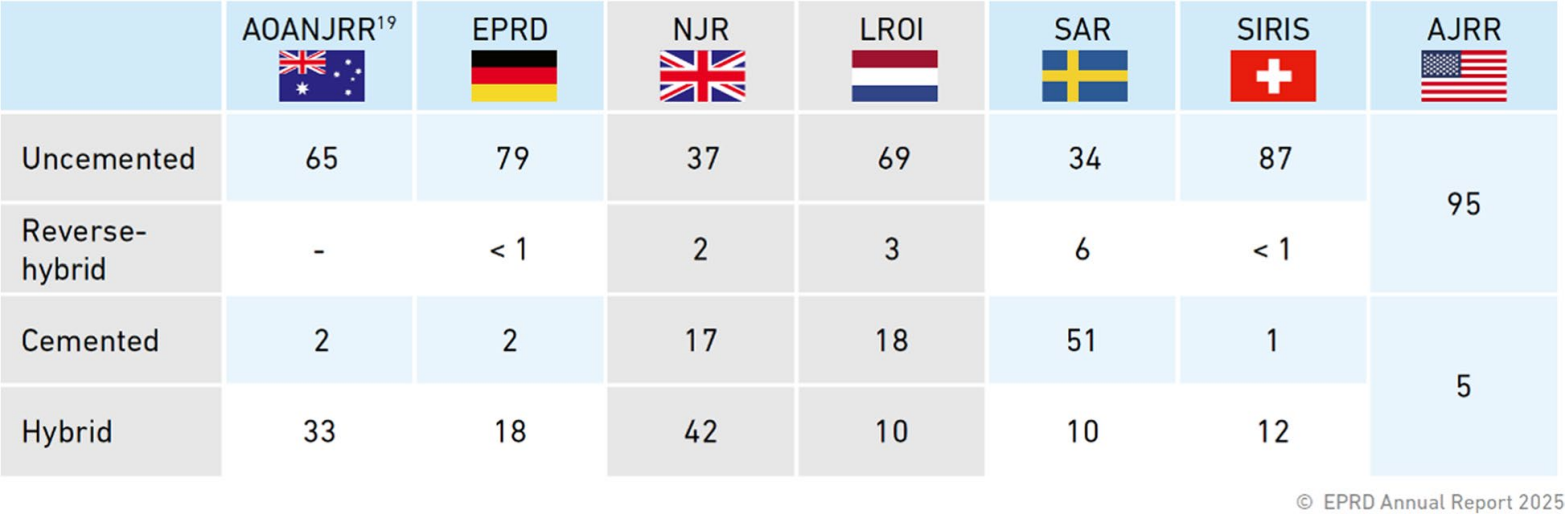
Percentage of fixation techniques used in total hip arthroplasty in the international endoprosthetic registries. Source: German arthroplasty registry annual report 2025, Table 68. [9] *Estimated values, from graphs in the annual report (Data for 2023 applies to “Metropolitan Centres”)

Total hip arthroplasty (THA) reliably restores pain-free function and quality of life, with long-term survivorship reported across multiple registries [1, 2]. Due to these results, it is often regarded as one of the most successful operations of the twentieth century [3]. In the past decades, the operative technique, especially the cementation technique, has evolved notably, with most recently the third-generation cementation technique being the gold standard [4]. Third-generation protocols involve pulsed lavage of the bone, femoral bone canal preparation with a distal restrictor, reaming of the acetabulum with dedicated attention to the Charnley zone 1, vacuum mixing and pressurization of the cement. When respecting these techniques, a more favorable outcome can be achieved [5, 6]. Cementless fixation, on the other hand, is commonly favored in younger patients due to the high capability of biological, secondary osseointegration of the implants, which yields excellent mid- to long-term survivorship [7]. In older patients (> 75-year-old), there is widespread use of cementless acetabular components combined with cemented stems (“hybrid” fixation of THA), with data suggesting that femoral-sided revisions are significantly higher in the fully uncemented THA in this age group [8]. A contemporary international endoprosthetic registry comparison (Fig. 1) indicates that the majority of primary THAs were performed without cemented fixation of the cup component [9]. The only exception to this predominantly cementless trend is Sweden, with 59% of its cup components being implanted with cemented fixation. In contrast, Germany had a total of < 3% of total hip arthroplasties with a cemented cup component.

Looking in more detail at one of the registries mentioned above, the Australian Orthopaedic Association National Joint Replacement Registry (AOANJRR) of 2024 demonstrates that the cumulative revision rates of primary total hip arthroplasties show no significant differences at a 3.5 + year follow-up for cementless vs. hybrid vs. cemented fixation techniques [10].

When taking a closer look at the data and differentiating between young patients (< 55-year-old) and older patients (≥ 75-year-old), a non-significant improved long-term outcome for cementless fixation in younger patients is observed. On the other hand, cemented and hybrid fixation in older patients has significantly lower revision rates (*p* < 0.001, respectively) compared to cementless fixation [10]. The trend of favorable outcomes of cemented fixation in older patients from the AOANJRR data is substantiated by a study from Jämsen et al. (2014), which analyzed data of the Finnish arthroplasty registry, with cemented THA showing higher implant survival rates in octogenarian patients [11]. Evidence remains mixed regarding whether cemented versus cementless acetabular fixation confers superior early and mid-term outcomes in an elderly age group (≥ 75-year-old; ≥ 80-year-old) after risk-adjustment for implant design. The increasing number of THAs performed, combined with Germany’s ageing population, makes this a relevant topic [12].

Specific data from the German arthroplasty registry (EPRD) concerning cup fixation have not yet been reported and could yield interesting results which may have a substantial impact on the clinical treatment algorithm in ≥ 75- and ≥ 80-year-old patients. Therefore, given the high implant survival and low cumulative revision rates reported for cemented fixation in older patients across multiple international arthroplasty registries, this study aimed to compare cumulative revision rates after total hip arthroplasty according to acetabular fixation technique (cemented versus cementless) within the EPRD.

## Methods

A registry-based cohort study including a total of 330,910 THAs was conducted. All primary THAs recorded in the EPRD between 12 November 2012 and 31 March 2024 were included. The overall median follow-up was 3.48 years (IQR 1.52–5.82), with a mean follow-up of 3.79 years (SD 2.60) and a maximum follow-up duration of 11.38 years. The exact follow-up intervals for the individual cohorts are listed in Table 1. Data collection within the EPRD is based on three sources: (1) billing data from statutory health insurance providers, (2) a product database supplied by implant manufacturers, and (3) electronic case report forms submitted by participating hospitals. Linkage with statutory health insurance records enables longitudinal follow-up of patients, including revision procedures and mortality. The EPRD captures approximately 70% of all hip and knee arthroplasties performed in Germany and provides near-complete follow-up through insurer linkage. The total number of THAs performed was subdivided into the following groups due to differences in acetabular component and fixation technique: cementless, hemispherical cups; cemented polyethylene (PE) and cemented cross-linked polyethylene cups (XPE); threaded cups; cemented and cementless dual mobility cups. At this point, it should be noted that the EPRD does not distinguish between cemented vs cementless dual mobility cups. All data concerning these cups should therefore be interpreted with caution.

**Table 1:** Patient cohort characteristics depending on the acetabular component used during primary total hip arthroplasty in Germany

**Variable**	**Dual mobility** *N* = 8,868^*1*^	**Hemispherical** *N* = 281,344^*1*^	**Threaded** *N* = 19,839^*1*^	**PE** *N* = 13,880^*1*^	**XPE** *N* = 6,979^*1*^
**Age**					
< 75	3,189 (36%)	189,099 (67%)	12,074 (61%)	3,178 (23%)	1,602 (23%)
≥ 75	5,679 (64%)	92,245 (33%)	7,765 (39%)	10,702 (77%)	5,377 (77%)
< 80	4,755 (54%)	236,873 (84%)	16,074 (81%)	6,676 (48%)	3,254 (47%)
≥ 80	4,113 (46%)	44,471 (16%)	3,765 (19%)	7,204 (52%)	3,725 (53%)
**ASA Score**					
ASA < 3	2,340 (39%)	93,643 (69%)	3,378 (64%)	1,768 (46%)	1,158 (43%)
ASA ≥ 3	3,703 (61%)	42,629 (31%)	1,860 (36%)	2,076 (54%)	1,562 (57%)
Unknown	2,825	145,072	14,601	10,036	4,259
**BMI**					
< 18.5	266 (3.0%)	1,979 (0.7%)	150 (0.8%)	212 (1.5%)	132 (1.9%)
18.5–24.99	3,126 (35%)	59,842 (21%)	3,476 (18%)	3,118 (22%)	2,063 (30%)
25.0–29.99	2,573 (29%)	82,690 (29%)	4,644 (23%)	3,074 (22%)	1,944 (28%)
30.0–34.99	1,254 (14%)	46,099 (16%)	2,601 (13%)	1,374 (9.9%)	800 (11%)
35.0–39.99	461 (5.2%)	17,069 (6.1%)	933 (4.7%)	411 (3.0%)	219 (3.1%)
≥ 40	231 (2.6%)	6,908 (2.5%)	373 (1.9%)	135 (1.0%)	101 (1.4%)
Missing	957 (11%)	66,757 (24%)	7,662 (39%)	5,556 (40%)	1,720 (25%)
**Stem fixation**					
Cemented	5,329 (60%)	41,239 (15%)	4,465 (23%)	11,415 (82%)	5,655 (81%)
Cementless	3,511 (40%)	239,534 (85%)	15,366 (77%)	2,451 (18%)	1,324 (19%)
Unknown	28	571	8	14	0
**Type of THA**					
Elective THA	6,274 (71%)	267,058 (95%)	18,676 (94%)	11,306 (82%)	5,870 (84%)
Non-elective THA	2,571 (29%)	13,725 (4.9%)	1,155 (5.8%)	2,560 (18%)	1,109 (16%)
Unknown	23	561	8	14	0
**THA/year**					
≤ 200	3,452 (39%)	98,631 (36%)	7,171 (38%)	7,646 (56%)	3,344 (48%)
201–500	3,359 (38%)	94,490 (34%)	8,285 (43%)	4,086 (30%)	2,466 (36%)
> 500	2,019 (23%)	81,471 (30%)	3,665 (19%)	1,977 (14%)	1,121 (16%)
Unknown	38	6,752	718	171	48
**Elixhauser score**					
(≤ − 1)	790 (8.9%)	42,274 (15%)	2,841 (14%)	1,182 (8.5%)	584 (8.4%)
(0–3)	3,364 (38%)	176,089 (63%)	11,866 (60%)	6,381 (46%)	3,380 (48%)
(> 4)	4,714 (53%)	62,981 (22%)	5,132 (26%)	6,317 (46%)	3,015 (43%)
**Follow Up**					
Years, Mean (SD)	2.31 (1.99)	3.74 (2.57)	4.98 (2.71)	4.24 (2.79)	3.74 (2.53)
Median (Q1, Q3)	3.43 (1.49, 5.72)	1.82 (0.72, 3.41)	4.13 (1.79, 6.48)	5.19 (2.72, 7.20)	3.51 (1.56, 5.69)
Min, Max	0.00, 11.38	0.00, 10.70	0.00, 11.34	0.00, 11.20	0.00, 11.04

^*1*^n (%); *PE* polyethylene, *XPE* Cross-linked polyethylene, *ASA* American Society of Anesthesiologists, *BMI* body mass index, *THA* total hip arthroplasty, *THA/year* total amount of THA implanted in the hospital in one year

### Ethical statement

The EPRD has received a general institutional review board approval from the University of Kiel (approval number D 473/11). All patients included in the registry agree to the storage of their data and sign a declaration of consent.

### Statistical analysis

Descriptive statistics were used to summarize patient demographics and implant characteristics. Continuous variables are reported as means with standard deviations, while categorical variables are presented as absolute frequencies and percentages.

Patients were grouped according to acetabular cup design: dual mobility cups (*n* = 8,868), hemispherical press-fit cups (*n* = 281,344), threaded cementless cups (*n* = 19,839), cemented PE cups (*n* = 13,880), and XPE cups (*n* = 6,979).

Implant survival was assessed using cumulative revision rates for any reason. Revision was defined as the exchange or removal of any implant component. Follow-up time was calculated from the date of primary THA to the date of revision up to 8 years. Cumulative revision rates were estimated using Kaplan–Meier survival analysis and are reported with corresponding 95% confidence intervals calculated using the log–log method. Differences between groups were evaluated using log-rank tests. Predefined subgroup analyses were performed according to patient age (< 75-year-old vs. ≥ 75-year-old; < 80-year-old vs. ≥ 80-year-old), acetabular cup design, and femoral stem fixation (cemented vs. cementless). Furthermore, the association between acetabular cup construct and revision risk was assessed using multivariable Cox proportional hazards regression models. Models were adjusted for sex, stem fixation, age group (< 75-year-old vs. ≥ 75-year-old; < 80-year-old vs. ≥ 80-year-old), Elixhauser comorbidity score, BMI category, annual hospital procedure volume (THA/year), and procedure type (elective vs. non-elective). Because of a high proportion of missing ASA classifications, the Elixhauser comorbidity score was used for comorbidity adjustment. Prespecified subgroup analyses were conducted according to the categories described in the manuscript. Adjusted hazard ratios (aHRs) and 95% confidence intervals (CIs) are reported.

A *p*-value < 0.05 was considered statistically significant. All statistical analyses were conducted using R version 4.4.2 (R Foundation for Statistical Computing, Vienna, Austria).

## Results

This study includes a total of 330,910 total hip arthroplasties performed in Germany. The demographic data of the total collective is shown in Table 1.

**Fig. 2:**
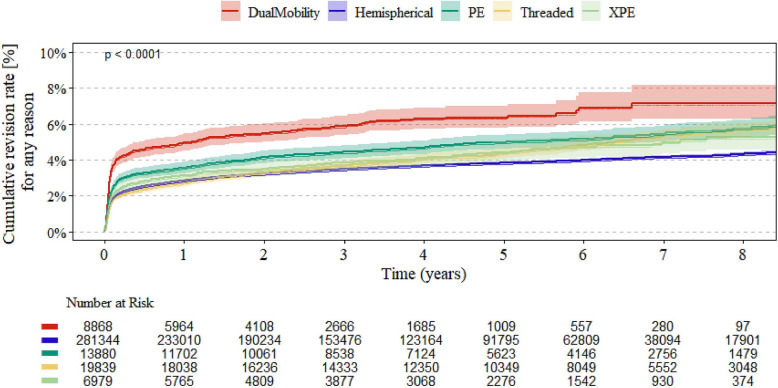
Estimated cumulative revision rates at eight years for any reason for different cup types in total hip arthroplasty in Germany in a cohort of *n* = 330,910 patients. In this analysis, no adjustment for femoral stem fixation was performed

In the total cohort, for the cementless hemispherical press-fit cup, a cumulative revision rate of 4.3% at eight years was observed, which was significantly lower (*p* < 0.001) than the other groups. In contrast to this, cemented PE and XPE cups showed a revision rate of 5.7% and 5.3%, respectively. Threaded cups had a revision rate of 5.7%, while dual mobility cups had the highest estimated cumulative revision rate of 7.2% cumulative revision rate at eight years. These results are highlighted in Fig. 2.

**Fig. 3:**
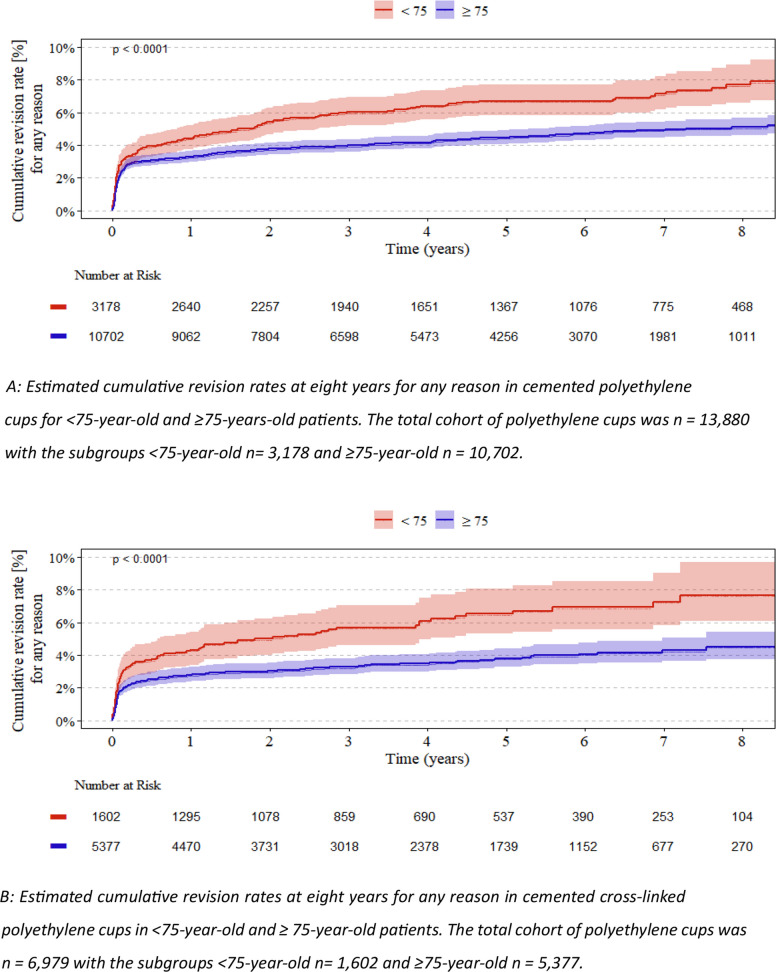
Estimated cumulative revision rates at eight years for any reason. **A** Cemented polyethylene cups in patients aged < 75 years and ≥ 75 years. The total cohort comprised 13,880 cups, including 3,178 in patients aged < 75 years and 10,702 in patients aged ≥ 75 years. **B** Cemented cross-linked polyethylene cups in patients aged < 75 years and ≥ 75 years. The total cohort comprised 6,979 cups, including 1,602 in patients aged < 75 years and 5,377 in patients aged ≥ 75 years

Cemented PE cups (*n* = 13,880) had an estimated eight-year cumulative revision rate for any reason of 7.7% in < 75-year-old patients and 5.1% in ≥ 75-year-old patients (*p* < 0.0001) (Fig. 3A). The same analysis was performed for XPE cups (*n* = 6,979) yielding the result of an estimated eight-year cumulative revision rate of 7.7% in < 75-year-old patients and a 4.5% cumulative revision rate in ≥ 75-year-old patients (*p* < 0.0001) (Fig. 3B).

**Fig. 4:**
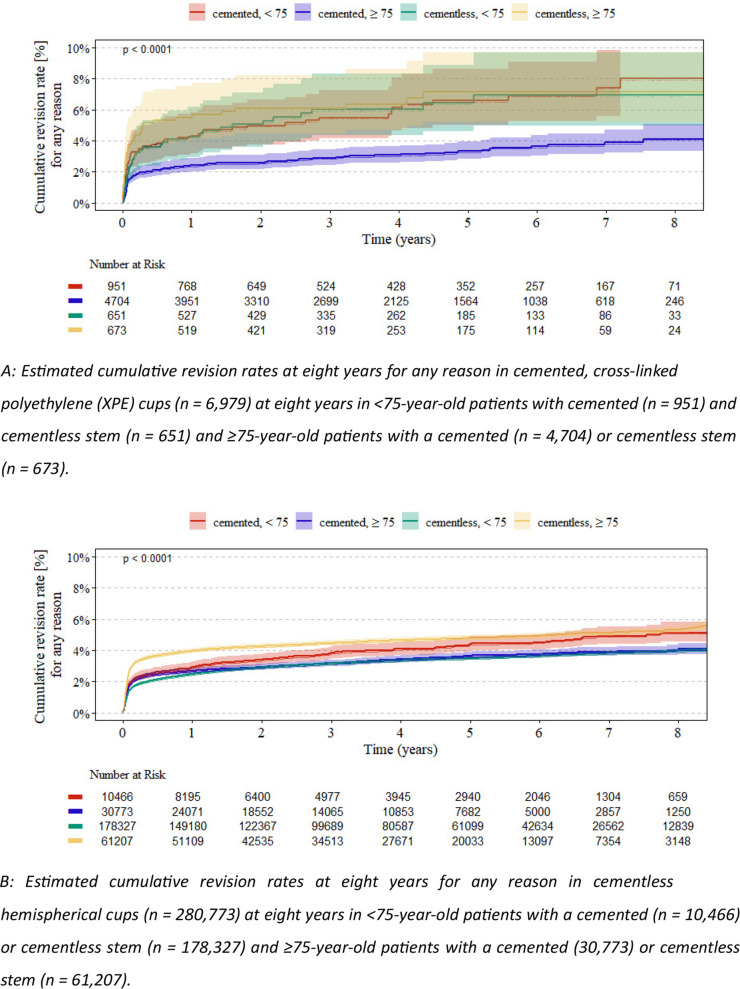
Estimated cumulative revision rates at eight years for any reason, stratified by patient age and stem fixation. **A** Cemented cross-linked polyethylene (XPE) cups (*n* = 6,979): patients aged < 75 years with a cemented stem (*n* = 951) or cementless stem (*n* = 651), and patients aged ≥ 75 years with a cemented stem (*n* = 4,704) or cementless stem (*n* = 673). **B** Cementless hemispherical cups (*n* = 280,773): patients aged < 75 years with a cemented stem (*n* = 10,466) or cementless stem (*n* = 178,327), and patients aged ≥ 75 years with a cemented stem (*n* = 30,773) or cementless stem (*n* = 61,207)

To evaluate this relationship further, we analyzed the cumulative revision rate of cemented PE and XPE cups not only depending on the age but also on stem fixation used in total hip arthroplasty. Within the further subgroup of ≥ 75-year-old patients, with an XPE cup and a cemented stem (*n* = 4,704), we found the lowest estimated cumulative revision rate of 4.1% at eight years after surgery (Fig. 4A). This survivorship analysis is equal to the cumulative revision rate of hybrid THA for ≥ 75-year-old patients (Fig. 4B). In contrast to this, cementless THA in < 75-year-old patients had the lowest estimated cumulative revision rate of 3.8% at eight years.

**Fig. 5:**
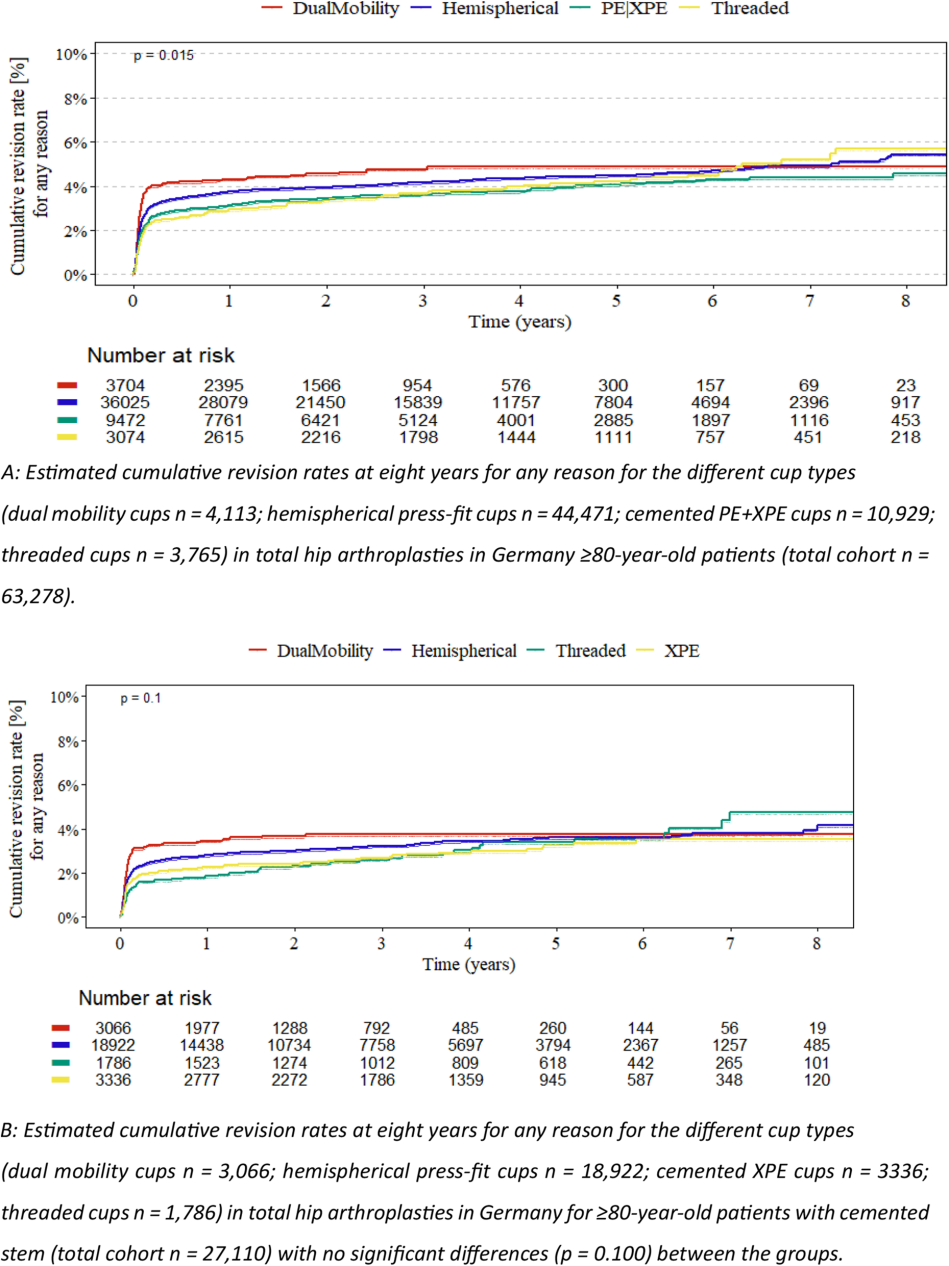
Estimated cumulative revision rates at eight years for any reason according to cup type in patients aged ≥ 80 years undergoing total hip arthroplasty in Germany. **A** The total cohort comprised 63,278 patients: dual-mobility cups (*n* = 4,113), hemispherical press-fit cups (*n* = 44,471), cemented polyethylene and cross-linked polyethylene (PE + XPE) cups (*n* = 10,929), and threaded cups (*n* = 3,765). **B** Among patients with a cemented stem, the total cohort comprised 27,110 patients: dual-mobility cups (*n* = 3,066), hemispherical press-fit cups (*n* = 18,922), cemented XPE cups (*n* = 3,336), and threaded cups (*n* = 1,786). No significant differences were observed between the groups (*p* = 0.100)

When looking at ≥ 80-year-old patients (with cementless and cemented stem fixation), a different outcome was observed. This collective consisted of *n* = 63,278 patients (*n* = 10,929 cemented cups; *n* = 52,349 cementless cups). Here, the group of cemented (X)PE cups had the lowest estimated cumulative revision rate after eight years, with 4.5% (*p* = 0.015) (Fig. 5A). The other acetabular fixation methods had the following estimated cumulative revision rates: hemispherical press-fit cups (5.4%), dual mobility cups (4.9%), and threaded cups (5.7%). When analyzing exclusively XPE cups, the total estimated cumulative revision rate drops to 4.1% at eight years. These results were analyzed independently of the femoral fixation method.

In ≥ 80-year-old patients with a cemented femoral stem, the lowest estimated cumulative revision rate was found in XPE cups with no significant differences at 8 years between the groups (*p* = 0.100) (Fig. 5B).

This analysis was able to identify the following implant- and patient-specific factors that significantly affect revision-free survival in cemented (X)PE cups:

Bigger femoral head size was associated with significantly lower (*p* < 0.0001) estimated cumulative revision rates of 4.8% in 36 mm, 5.5% in 32 mm, and 8.1% in 28 mm at eight years, respectively. Furthermore, ceramic on polyethylene (CoP) had significantly lower estimated cumulative revision rates (*p* < 0.0001) than the metal on polyethylene (MoP) bearing surface combination (5.2% vs. 6.4%) at eight years. Patients with an ASA score of ≥ 3 performed significantly worse (*p* < 0.0001) than patients with an ASA score of < 3 (6.2% vs. 3.6%). Lastly, a BMI of ≤ 30 was associated with a significantly lower (*p* < 0.0001) estimated cumulative rate in comparison with patients with a BMI of > 30 (4.9% vs. 7.5%).

### Multivariable cox proportional hazards regression model

When conducting a multivariable Cox proportional hazards regression model, the overall results of XPE cup revision risk were comparable to those of hemispherical cups. Overall, XPE cups showed no significant differences compared to hemispherical press-fit cups (aHR = 1.10, 95% CI 0.98–1.25, *p* = 0.120).

For patients younger than 75 years, multivariable Cox regression analysis identified that, compared with hemispherical cups, the risk of revision was significantly higher for dual mobility (aHR 1.68, 95% CI 1.45–1.95, *p* < 0.001), PE (aHR 1.50, 95% CI 1.28–1.74, *p* < 0.001), Threaded (aHR 1.26, 95% CI 1.15–1.37, *p* < 0.001), and XPE cups (aHR 1.51, 95% CI 1.22–1.86, *p* < 0.001).

For patients aged 75 years or older, a tendency towards, but not significant, risk reduction was observed for XPE cups (aHR = 0.98, 95% CI 0.84–1.14, *p* = 0.800). The same was observed for patients aged 80 years or older. Here, a non-significant risk reduction was observed (aHR = 0.98, 95% CI 0.81–1.19, *p* = 0.800). A similar result was reported for patients with a cemented stem and XPE cups compared to patients with a cemented stem and hemispherical cups (aHR = 0.99, 95% CI 0.85–1.15, *p* = 0.900).

## Discussion

### Cohort and data

This study analyzed a representative cohort of 330,910 patients from the EPRD. As Germany is a high-income country in which total joint arthroplasty is routinely performed under very high technical and organizational standards, the registry population reflects contemporary practices with high standards. The nationwide coverage of the EPRD ensures inclusion of a broad spectrum of treating institutions and patient profiles, thereby enhancing the external validity and generalizability of our findings. Furthermore, the follow-up period of on average 3.8 years (with a maximum of 11.38 years) allows for a robust assessment of midterm outcomes, capturing both early and later adverse events and revision procedures for cup fixation in total hip arthroplasty.

### Cup fixation

In the context of total hip arthroplasty, the choice of acetabular cup fixation remains clinically relevant because detailed cumulative revision rates by fixation type are still underrepresented in the literature, despite evidence that fixation strongly modifies implant longevity. Large-scale analyses are difficult to obtain at the single-center level, as modern practice encompasses multiple fixation strategies. These include cementless hemispherical press-fit cups, threaded cups, cemented (X)PE cups, and dual-mobility constructs. To further complicate matters, each fixation technique is predominantly employed in different patient populations, making it difficult to generate large, comparable cohorts. In our nationwide cohort of 330,910 primary THAs from the EPRD, hemispherical cementless cups represented by far the largest subgroup (*n* = 281,344), and this group contained a disproportionately high share of younger patients (67% < 75-year-old; 84% < 80-year-old), whereas cemented PE/XPE cups (*n* = 20,859) were preferentially implanted in older individuals (77% ≥ 75-year-old; 52% ≥ 80-year-old). Overall, the lowest estimated cumulative revision rates for any reason were achieved by the hemispherical press fit cups (with cementless and cemented stem fixation), as shown in Fig. 2. However, the XPE cup showed no significantly higher revision risk in multivariable regression analysis, whereas the other cup fixation methods had a significantly higher revision risk. This age imbalance introduces a relevant statistical bias when crude cumulative revision rates are compared across fixation types. After age stratification, however, a consistent pattern in Kaplan–Meier analysis emerges in < 75-year-old patients: cementless hemispherical cups show lower 8-year Kaplan–Meier estimated cumulative revision rates (4.2%), compared to dual-mobility cups (8.4%), cemented PE/XPE cups (6.3%), and threaded cups (5.7%). Additionally, multivariable regression showed a consistent pattern for patients < 75 years old with increased revision risk in every cup fixation method compared to hemispherical press-fit cups. This reinforced the usage of cementless hemispherical press-fit fixation cups in < 75-year-old patients. This finding is in line with registry data on contemporary press-fit cups, which report survival rates of around 95% at 10 years and 91% at 18 years in predominantly younger, cementless cohorts [13, 14]. In contrast, among ≥ 80-year-old patients, descriptive survival analyses suggested a different pattern: at 8 years, cemented PE/XPE cups exhibit the lowest estimated cumulative revision rate (4.5%), slightly lower than dual-mobility designs (4.9%) and clearly lower than cementless hemispherical (5.4%) and threaded cups (5.7%). These findings may indicate that the relative performance of cup designs differs in very elderly patients compared to younger populations. However, it must be noted that after adjustment for potential confounders in multivariable Cox regression models, these differences were not statistically significant, suggesting that the observed differences in unadjusted revision rates should be interpreted with caution.

Comparable age-dependent effects of cup fixation are reported from Scandinavian and Danish joint registries: in a combined Nordic database, 10-year survival of cemented implants in patients aged 65–74 and ≥ 75-year-old patients (93.8% and 95.9%) is higher than that of uncemented and hybrid constructs (92.9% and 92.3%), whereas survival is similar between cemented and uncemented THA in patients 55–64 years old [15]. Analyses from the Danish Hip Arthroplasty Register show an increased risk of revision for uncemented and hybrid THA compared to cemented cups, particularly in older age groups [16]. The Swedish Hip Arthroplasty Register data indicate a higher risk of cup revision due to aseptic loosening for uncemented versus cemented cups, despite improvements in newer uncemented designs [17]. The AOANJRR data demonstrates excellent long-term survivorship of cemented acetabular components, with low cumulative percent revision out to 15 years, especially when implanted by high-volume surgeons [18]. Taken together, our analysis complements national registry findings by providing fixation-specific, age-stratified cumulative revision estimates. Consistent with reports from the Scandinavian, Danish, Swedish, and Australian registries, descriptive survival analyses favored cemented cups in elderly patients. However, after adjustment for relevant confounders, no statistically significant differences in revision risk were observed, suggesting that the apparent survival advantage may be partly attributable to differences in patient selection and case mix. Nevertheless, our findings indicate that cemented acetabular fixation remains a reliable option in elderly patients.

### Risks and reasons against cemented cups

Cemented acetabular components in THA are associated with several procedure-specific risks that may argue against their routine use. The use of polymethylmethacrylate introduces the possibility of bone cement implantation syndrome (BCIS), a spectrum of embolic and cardiopulmonary events characterized by intraoperative hypoxia, hypotension, and, in severe cases, cardiovascular collapse [19]. A study by Schwarzkopf et al. (2019) has observed that BCIS is associated with an increased incidence and mortality in patients > 60-year-old, ASA III–IV patients with oncologic comorbidities, and those with relevant cardiopulmonary comorbidities [20]. This is relevant because these patients are exactly the cohort where a cemented cup seems to be justified. However, the risk of BCIS is primarily given during the implantation of the femoral component and can be modulated by performing correct, third-generation cementing techniques as described by Clarius et al. (2005) [21]. These techniques include the obligatory pulsatile lavage of the bone to reduce fat and bone marrow intravasation and correct acetabular preparation [22, 23]. In addition, cemented cups carry the specific risk of intrapelvic extrusion of cement through a compromised medial wall. Case series and reports have described delayed migration or fragmentation of intrapelvic cement masses causing bladder perforation, gross hematuria, ureteric obstruction with hydronephrosis, and injury to pelvic vessels or nerves (e.g., obturator or lumbosacral plexus), frequently requiring complex revision for removal [24–26]. This complication profile contrasts with that of hemispherical press-fit cups, where the dominant concern is intraoperative, periprosthetic acetabular fracture during reaming or shell impaction [27]. Finally, cemented acetabular fixation generally prolongs operative time, and therefore indirect costs, because of the additional steps required for 3rd-generation cementation. A review by Van Praet et al. (2019) reported an additional time of 15–20 min per case when cemented components are used instead of cementless ones [28]. Here, the cement setting time must be considered. It depends on the temperature of the bone cement and the manufacturer and varies between 10 and 12 min, followed by the additional steps for correct cementation and for reduction of BCIS. This brings the total additional time in comparison to hemispherical press-fit cups up to the above-mentioned estimates of 15–20 min. However, it should be mentioned that these times can be optimized in a high-volume setting with standardized protocols. This leads to an indirect increase in the cost per surgery.

### Patient and implant-specific factors

Established determinants that affect revision-free survival in THA were confirmed, particularly femoral head size and the bearing surface combination. Larger femoral heads were associated with lower revision rates (*p* < 0.0001). In addition, CoP bearing couple demonstrated significantly higher revision-free survival than MoP (*p* < 0.0001), with lower cumulative revision at eight years (5.2% vs. 6.4%). These findings align with prior evidence that both head diameter and bearing surface combination can influence mechanical stability, wear-related mechanisms, and ultimately implant survival in cemented (X)PE cups [29–32]. Kostensalo et al. (2013) showed in a registry-based analysis that larger femoral heads were associated with a significantly lower revision risk for dislocation after THA, highlighting improved joint stability as a key mechanism [29]. Similarly, Matar et al. (2024), in a comparative study of more than 10,000 hips, reported significantly lower revision rates with 32 mm and 36 mm femoral heads compared with 28 mm heads, further supporting the protective effect of larger head sizes against revision [30]. Regarding wear, Whitehouse et al. (2024), analyzing more than one million primary THAs, demonstrated that bearing surface selection significantly influences revision risk, with ceramic-based articulations showing favorable long-term outcomes compared with conventional metal-on-polyethylene constructs [31]. Likewise, Davis et al. (2020) reported that bearing surface combinations affected survivorship in both cementless and hybrid THAs, with ceramic-on-polyethylene bearings associated with improved implant survival and lower revision rates compared with metal-on-polyethylene bearings [32].

Regarding patient-related factors, ASA score and BMI showed strong associations with outcome and yielded clinically relevant differences. Patients with an ASA score of ≥ 3 had significantly worse revision-free survival than those with an ASA score of < 3 (6.2% vs. 3.6%; *p* < 0.0001). Similarly, a BMI ≤ 30 was associated with a significantly better outcome compared with BMI > 30 (4.9% vs. 7.5%; *p* < 0.0001). Importantly, these associations should be interpreted as reflecting a general relationship between overall patient risk profile and implant survival, rather than implying a mechanism unique to cemented (X)PE cups. An ASA score of ≥ 3 class captures systemic comorbidity relevant to surgical procedures that can increase the risk for complications and reoperation and therefore affect revision-free survival, examined in this analysis [33–35]. An elevated BMI not only increases implant wear, but also generally increases the risk for wound healing disorders, infection, and subsequently reoperation, affecting implant survivorship [36–38]. Consequently, these variables are best viewed as broad, patient-level prognostic markers within this cemented cup cohort, rather than as factors specific to the cemented (X)PE cup.

### Cost

Internationally, cemented XPE cups with the necessary cement are typically cheaper than cementless hemispherical (press-fit) cups with the corresponding inlay. In the literature, typical published implant-only cost ranges are 1,020–1,800 € for cemented fixation vs. 1,800–4,040 € for cementless fixation [39, 40]. The cost of the hemispherical press-fit cups has a higher variability due to different inlay choices and modular components that can be added to the cup. Furthermore, the porous coating that facilitates osseointegration is priced higher by manufacturers.

### Illustrative scenario in Germany

An exemplary case in Germany would cost 110–300 € for an XPE cup and bone cement. A typical cementless cup fixation would cost 480–650 € (for the shell and an additional XPE inlay). During the period (11.38 years) that was analyzed in this study, 44,471 hemispherical cups were implanted in ≥ 80-year-old patients in Germany, which means that a considerable amount of money (approximately 16,000,000 € when calculating with average implant cost differences) could have been saved if using the cheaper cemented XPE cups. Even when accounting for an, on average, prolonged operative time of 15 min per case with a cost of 18.4 €/min (approximately 12,200,000 €), an estimated 3.8 million € could have been saved by using the cheaper implant, which has no significant differences in Cox proportional hazard regression in elderly patients compared to hemispherical press fit cups. Given the competing risk of mortality in the very elderly patients (≥ 80 years old), these results should be interpreted with caution and are only reported for illustrative purposes.

The revision-free survival observed for dual-mobility constructs and threaded acetabular cups in this study should be interpreted with caution. First, these subgroups were relatively small compared to the (X)PE and hemispherical group, limiting statistical power and yielding wider uncertainty around survival estimates. Second, this analysis could not adjust for the indication of the implant choice. Also, the registry does not differentiate between cemented and cementless dual mobility cups. Dual mobility cups/liners and threaded cups can be employed in complex situations such as acetabular bone loss and instability risk that can affect the outcome [41, 42]. Therefore, a part of the effect observed in this study may reflect selection bias for the respective implant choice rather than an intrinsic performance advantage or disadvantage of the implant design.

### Limitations

As an observational registry-based analysis, confounding by indication cannot be excluded, as specific implants such as dual mobility cups may have been preferentially used in elderly or high-risk patients. Although the EPRD provides high-quality nationwide data, it captures approximately 70% of arthroplasties performed in Germany and can sometimes provide incomplete data for certain variables (particularly ASA status) when the information is not provided correctly, which may limit adjustment for relevant comorbidities. Cases with incomplete data were excluded from subgroup analysis, which is an important selection bias and limits the results from subgroup analysis. Furthermore, registry data are derived from billing records, manufacturer product databases, and electronic case report forms. This is a potential risk for coding inaccuracies, misclassification of implant types, and the absence of radiographic or clinical validation.

As mortality poses a significant competing event for implant revision in an elderly cohort, particularly in ≥ 80-year-old patients, the absence of a competing risk analysis is a major methodological limitation which may have led to an overestimation of revision rates in these cohorts.

## Conclusion

Although cementless hemispherical press-fit cups are, in every age category, the most popular cup option for THA in Germany, the data from the German national registry reinforce the use of cemented XPE cups for certain subgroups.

In < 75-year-old patients, all alternative cup designs, including XPE cups, were associated with a significantly increased risk of revision compared with hemispherical press-fit cups. In contrast, among ≥ 75-year-old patients, no significant differences in revision risk were observed between XPE and hemispherical cups. These findings suggest that the relative advantage of hemispherical press-fit cups observed in younger patients diminishes with increasing age and is no longer evident in elderly patients.

Independent of age and femoral fixation, cemented (X)PE cup survivorship benefits significantly from CoP bearing surface combination and bigger head size (36 mm > 32 mm > 28 mm).

Higher BMI and greater comorbidity burden were associated with an increased revision risk in the adjusted analyses.

Furthermore, in the 11.38 years analyzed in this study, a total of *n* = 44,471 hemispherical press-fit cups were implanted in ≥ 80-years-old patients, indicating potential for cost savings when opting for the cheaper implant, which offers comparable results (no significant differences in multivariable Cox proportional hazard regression for XPE vs. hemispherical cups in ≥ 75- and ≥ 80-year-old patients). However, these findings should be interpreted with caution, as the competing risk of mortality in very elderly patients may influence the observed revision rates.

These findings have important clinical implications for implant selection in elderly patients undergoing THA, suggesting that age-specific fixation strategies should be considered rather than a uniform preference for cementless acetabular fixation. In particular, cemented XPE cups may represent a valuable option for ≥ 75-year-old patients, especially in octogenarians, from a survivorship perspective and may pose health-economic benefits and cost-saving opportunities. Future studies should focus on prospective analyses of patient-reported outcomes, complication profiles, and long-term cost-effectiveness for acetabular fixation in THA to further define the optimal fixation strategy for different age and risk groups.

## Data Availability

The data supporting the findings of this study are included in the article. Further inquiries may be directed to the German Arthroplasty Registry. The German Arthroplasty Registry can be contacted by email at info@eprd.de.

## Abbreviations

THA: Total hip arthroplasty
AOANJRR: Australian Orthopaedic Association National Joint Replacement Registry
EPRD: German Arthroplasty Registry
PE: Cemented polyethylene
XPE: Highly cross-linked polyethylene
ASA: American Society of Anesthesiologists
BMI: Body mass index
CoP: Ceramic on polyethylene
MoP: Metal on polyethylene
BCIS: Bone cement implantation syndrome
aHR: Adjusted hazard ratio
CI: Confidence interval

## References

[1] Shan L, Shan B, Graham D, Saxena A. Total hip replacement: a systematic review and meta-analysis on mid-term quality of life. Osteoarthritis Cartilage. 2014;22(3):389–406. 10.1016/j.joca.2013.12.006. 24389057 10.1016/j.joca.2013.12.006

[2] Rolfson O, Kärrholm J, Dahlberg LE, Garellick G. Patient-reported outcomes in the Swedish Hip Arthroplasty Register. J Bone Joint Surg Br. 2011;93-B(7):867–75. 10.1302/0301-620X.93B7.25737 10.1302/0301-620X.93B7.25737 21705555

[3] Learmonth ID, Young C, Rorabeck C. The operation of the century: total hip replacement. The Lancet. 2007;370(9597):1508–19. 10.1016/S0140-6736(07)60457-7. 10.1016/S0140-6736(07)60457-7 17964352

[4] Breusch S, Malchau H. The well-cemented total hip arthroplasty. Berlin, Heidelberg: Springer Berlin Heidelberg; 2005. 10.1007/3-540-28924-0.

[5] Humez M, Fröschen FS, Wirtz DC, Kühn KD. Moderne Zementiertechnik der dritten Generation in der Knie- und Hüftendoprothetik. Die Orthopädie. 2023;52(12):968–80. 10.1007/s00132-023-04446-7. 37828239 10.1007/s00132-023-04446-7

[6] Hirose S, Otsuka H, Morishima T, Sato K. Outcomes of Charnley total hip arthroplasty using improved cementing with so-called second- and third-generation techniques. J Orthop Sci. 2012;17(2):118–23. 10.1007/S00776-011-0180-X. 22189995 10.1007/s00776-011-0180-x PMC3314183

[7] Wechter J, Comfort TK, Tatman P, Mehle S, Gioe TJ. Improved Survival of Uncemented versus Cemented Femoral Stems in Patients Aged < 70 Years in a Community Total Joint Registry. Clin Orthop Relat Res. 2013;471(11):3588–95. 10.1007/s11999-013-3182-5. 23873609 10.1007/s11999-013-3182-5 PMC3792261

[8] Boyle AB, Zhu M, Frampton C, Poutawera V, Vane A. Comparing modern uncemented, hybrid and cemented implant combinations in older patients undergoing primary total hip arthroplasty, a New Zealand Joint Registry study. Arch Orthop Trauma Surg. 2022;143(6):3597–604. 10.1007/s00402-022-04610-2. 36102955 10.1007/s00402-022-04610-2

[9] Endoprothesenregister Deutschland (EPRD). EPRD-Jahresbericht 2025. Berlin: EPRD Deutsche Endoprothesenregisterg GmbH; 2025. 10.36186/reporteprd122025. Available from: https://www.eprd.de/fileadmin/user_upload/Dateien/Publikationen/Berichte/Jahresbericht2025-Status5_2025-10-28_F.pdf.

[10] Lewis PL, Gill DR, McAuliffe MJ, McDougall C, Stoney JD, Vertullo CJ, et al. Lay Summary: Hip. Knee and Shoulder Replacement. 2024. 10.25310/FMSU7258.

[11] Jämsen E, Eskelinen A, Peltola M, Mäkelä K. High Early Failure Rate After Cementless Hip Replacement in the Octogenarian. Clin Orthop Relat Res. 2014;472(9):2779–89. 10.1007/s11999-014-3641-7. 24771260 10.1007/s11999-014-3641-7 PMC4117887

[12] Rupp M, Lau E, Kurtz SM, Alt V. Projections of Primary TKA and THA in Germany From 2016 Through 2040. Clin Orthop Relat Res. 2020;478(7):1622–33. 10.1097/CORR.0000000000001214. 32168057 10.1097/CORR.0000000000001214 PMC7310374

[13] Lunz A, Von Falkenhayn M, Jaeger S, Reiner T, Merle C, Streit MR, et al. Minimum 20-year follow-up of a press-fit acetabular cup in cementless total hip replacement in young patients. Acta Orthop. 2023;4:94. 10.2340/17453674.2023.13385. 10.2340/17453674.2023.13385 PMC10326741 37409417

[14] Hailer NP, Garellick G, Kärrholm J. Uncemented and cemented primary total hip arthroplasty in the Swedish Hip Arthroplasty Register. Acta Orthop. 2010;81(1):34–41. 10.3109/17453671003685400. 20180715 10.3109/17453671003685400 PMC2856202

[15] Makela KT, Matilainen M, Pulkkinen P, Fenstad AM, Havelin L, Engesaeter L, et al. Failure rate of cemented and uncemented total hip replacements: register study of combined Nordic database of four nations. BMJ. 2014;348(jan13 12):f7592–f7592. 10.1136/bmj.f7592. 24418635 10.1136/bmj.f7592

[16] Pedersen AB, Mehnert F, Havelin LI, Furnes O, Herberts P, Kärrholm J, et al. Association between fixation technique and revision risk in total hip arthroplasty patients younger than 55 years of age. Results from the Nordic Arthroplasty Register Association. Osteoarthritis Cartilage. 2014;22(5):659–67. 10.1016/j.joca.2014.03.005. 24631923 10.1016/j.joca.2014.03.005

[17] Hailer NP, Garellick G, Kärrholm J. Uncemented and cemented primary total hip arthroplasty in the Swedish Hip Arthroplasty Register. Acta Orthop. 2010;81(1):34–41. 10.3109/17453671003685400. 20180715 10.3109/17453671003685400 PMC2856202

[18] Hanly RJ, Whitehouse SL, Lorimer MF, de Steiger RN, Timperley AJ, Crawford RW, et al. The outcome of cemented acetabular components in total hip arthroplasty for osteoarthritis defines a proficiency threshold: results of 22,956 cases from the Australian Orthopaedic Association National Joint Replacement Registry. J Arthroplasty. 2019;34(8):1711–7. 10.1016/j.arth.2019.03.061. 31031154 10.1016/j.arth.2019.03.061

[19] Donaldson AJ, Thomson HE, Harper NJ, Kenny NW. Bone cement implantation syndrome. Br J Anaesth. 2009;102(1):12–22. 10.1093/bja/aen328. 19059919 10.1093/bja/aen328

[20] Schwarzkopf E, Sachdev R, Flynn J, Boddapati V, Padilla RE, Prince DE. Occurrence, risk factors, and outcomes of bone cement implantation syndrome after hemi and total hip arthroplasty in cancer patients. J Surg Oncol. 2019;120(6):1008–15. 10.1002/jso.25675. 31432531 10.1002/jso.25675 PMC7357733

[21] Michael Clarius CHSJB. Pulmonary embolism in cemented total hip arthroplasty. In: The well-cemented hip arthroplasty. Springer; 2005. p. 320–31.

[22] Breusch SJ, Reitzel T, Schneider U, Volkmann M, Ewerbeck V, Lukoschek M. Pulsatile lavage reduces the risk of fat embolism in cemented total hip arthroplasty. Orthopade. 2000;29(6):578–86. 10.1007/s001320050496. 10929338 10.1007/s001320050496

[23] Satalich JR, Lombardo DJ, Newman S, Golladay GJ, Patel NK. Cementation in total hip arthroplasty: history, principles, and technique. EFORT Open Rev. 2022;7(11):747–57. 10.1530/EOR-22-0002. 36475555 10.1530/EOR-22-0002 PMC9780613

[24] Lichtenberg AB, Ansari AZ, Bahro G, Bilal M, Lief S, Patibandla S, et al. Acetabular bone cement extension leading to bladder obstruction: an orthopedic surgical complication. Cureus. 2024. 10.7759/cureus.68694. 39371860 10.7759/cureus.68694 PMC11452919

[25] Thompson NW, Colleary G, Wilson DS, Crone MD, Beverland DE. Migration of intrapelvic cement after total hip arthroplasty. J Arthroplasty. 2002;17(3):382–3. 10.1054/arth.2002.30780. 11938519 10.1054/arth.2002.30780

[26] Greenspan A, Norman A. Gross hematuria: a complication of intrapelvic cement intrusion in total hip replacement. AJR Am J Roentgenol. 1978;130(2):327–9. 10.2214/ajr.130.2.327. 414587 10.2214/ajr.130.2.327

[27] Haidukewych GJ. Intraoperative fractures of the acetabulum during primary total hip arthroplasty. The Journal of Bone and Joint Surgery (American). 2006;88(9):1952. 10.2106/JBJS.E.00890. 10.2106/JBJS.E.00890 16951110

[28] Van Praet F, Mulier M. To cement or not to cement acetabular cups in total hip arthroplasty: a systematic review and re-evaluation. SICOT J. 2019;1(5):35. 10.1051/sicotj/2019032. 10.1051/sicotj/2019032 PMC6771226 31571579

[29] Kostensalo I, Junnila M, Virolainen P, Remes V, Matilainen M, Vahlberg T, et al. Effect of femoral head size on risk of revision for dislocation after total hip arthroplasty. Acta Orthop. 2013;84(4):342–7. 10.3109/17453674.2013.810518. 23799348 10.3109/17453674.2013.810518 PMC3768031

[30] Matar HE, van Duren BH, Bloch BV, Berber R, James PJ, Manktelow ARJ. Lower risk of revision with 32- and 36-millimeter femoral heads compared with 28-mm heads in primary total hip arthroplasty: a comparative single-center study (10,104 hips). J Arthroplasty. 2024;39(4):991–6. 10.1016/j.arth.2023.10.042. 38661490 10.1016/j.arth.2023.10.042

[31] Whitehouse MR, Patel R, French JMR, Beswick AD, Navvuga P, Marques EMR, et al. The association of bearing surface materials with the risk of revision following primary total hip replacement: a cohort analysis of 1,026,481 hip replacements from the National Joint Registry. PLoS Med. 2024;21(11):e1004478. 10.1371/journal.pmed.1004478. 39509352 10.1371/journal.pmed.1004478 PMC11542800

[32] Davis ET, Pagkalos J, Kopjar B. Effect of Bearing Surface on Survival of Cementless and Hybrid Total Hip Arthroplasty. JBJS Open Access. 2020;5(2):e0075–e0075. 10.2106/JBJS.OA.19.00075. 33123668 10.2106/JBJS.OA.19.00075 PMC7418917

[33] Hendrix JM, Garmon EH. American Society of Anesthesiologists Physical Status Classification System. [Updated 2025 Feb 11]. In: StatPearls [Internet]. Treasure Island (FL): StatPearls Publishing; 2026. Available from: https://www.ncbi.nlm.nih.gov/books/NBK441940/. 28722969

[34] Schaeffer JF, Scott DJ, Godin JA, Attarian DE, Wellman SS, Mather RC. The Association of ASA Class on Total Knee and Total Hip Arthroplasty Readmission Rates in an Academic Hospital. J Arthroplasty. 2015;30(5):723–7. 10.1016/j.arth.2014.12.014. 25575729 10.1016/j.arth.2014.12.014

[35] Ferguson RJ, Silman AJ, Combescure C, Bulow E, Odin D, Hannouche D, et al. ASA class is associated with early revision and reoperation after total hip arthroplasty: an analysis of the Geneva and Swedish Hip Arthroplasty Registries. Acta Orthop. 2019;90(4):324–30. 10.1080/17453674.2019.1605785. 31035846 10.1080/17453674.2019.1605785 PMC6718172

[36] Sayed-Noor AS, Mukka S, Mohaddes M, Kärrholm J, Rolfson O. Body mass index is associated with risk of reoperation and revision after primary total hip arthroplasty: a study of the Swedish Hip Arthroplasty Register including 83,146 patients. Acta Orthop. 2019;90(3):220–5. 10.1080/17453674.2019.1594015. 30931664 10.1080/17453674.2019.1594015 PMC6534237

[37] Onggo JR, Onggo JD, de Steiger R, Hau R. Greater risks of complications, infections, and revisions in the obese versus non-obese total hip arthroplasty population of 2,190,824 patients: a meta-analysis and systematic review. Osteoarthritis Cartilage. 2020;28(1):31–44. 10.1016/j.joca.2019.10.005. 31705995 10.1016/j.joca.2019.10.005

[38] Toh SMS, Ashkanfar A, English R, Rothwell G. The relation between body weight and wear in total hip prosthesis: a finite element study. Comput Methods Programs Biomed Update. 2022;2:100060. 10.1016/j.cmpbup.2022.100060.

[39] Veldman HD, de Bot RTAL, Heyligers IC, Boymans TAEJ, Hiligsmann M. Cost-effectiveness analyses comparing cemented, cementless, hybrid and reverse hybrid fixation in total hip arthroplasty: a systematic overview and critical appraisal of the current evidence. Expert Rev Pharmacoecon Outcomes Res. 2021;21(4):579–93. 10.1080/14737167.2021.1878880. 33472442 10.1080/14737167.2021.1878880

[40] Jameson SS, Mason J, Baker PN, Gregg PJ, Deehan DJ, Reed MR. Implant optimisation for primary hip replacement in patients over 60 years with osteoarthritis: a cohort study of clinical outcomes and implant costs using data from England and Wales. PLoS ONE. 2015;10(11):e0140309. 10.1371/journal.pone.0140309. 26561859 10.1371/journal.pone.0140309 PMC4643061

[41] Vajapey SP, Fideler KL, Lynch D, Li M. Use of dual mobility components in total hip arthroplasty: Indications and outcomes. J Clin Orthop Trauma. 2020;11(Suppl 5):S760–5. 10.1016/j.jcot.2020.07.035. 32999552 10.1016/j.jcot.2020.07.035 PMC7503159

[42] Pantè S, Fassino Vesconi R, Braconi L, D’Amelio A, Aprato A, Massè A. Threaded cup in total hip arthroplasty: a systematic review of long-term survivorship. Eur J Orthop Surg Traumatol. 2025;35(1):236. 10.1007/s00590-025-04364-8. 40473846 10.1007/s00590-025-04364-8

